# A childhood acute lymphoblastic leukemia-specific lncRNA implicated in prednisolone resistance, cell proliferation, and migration

**DOI:** 10.18632/oncotarget.13936

**Published:** 2016-12-15

**Authors:** Manon Ouimet, Simon Drouin, Mathieu Lajoie, Maxime Caron, Pascal St-Onge, Romain Gioia, Chantal Richer, Daniel Sinnett

**Affiliations:** ^1^ Division of Hematology-Oncology, Research Center, Sainte-Justine University Health Center, Montreal, QC, Canada; ^2^ Department of Pediatrics, University of Montreal, Montreal, QC, Canada

**Keywords:** long non-coding RNA, acute lymphoblastic leukemia, glucocorticoids, RNASeq, treatment resistance

## Abstract

Childhood acute lymphoblastic leukemia (cALL) is the most common pediatric cancer and, despite an 85% cure rate, still represents a major cause of disease-related death in children. Recent studies have implicated long non-coding RNAs (lncRNAs) in cALL etiology, progression, and treatment response. However, barring some exceptions little is known about the functional impact of lncRNAs on cancer biology, which limits their potential as potential therapeutic targets. We wanted to investigate the functional role of lncRNAs identified as specifically overexpressed in pre-B cALL by whole-transcriptome sequencing. Here we report five lncRNAs specifically upregulated in pre-B cALL that had significant impacts on cancer hallmark traits such as cell proliferation, migration, apoptosis, and treatment response. In particular, silencing of the *RP11-137H2.4* lncRNA effectively restored normal glucocorticoid (GC) response in a GC-resistant pre-B cALL cell line and specifically modulated expression of members of both the NRAS/BRAF/NF-?B MAPK cascade and cell cycle pathways. Since GC form the cornerstone of cALL chemotherapy and resistance in cALL confers a dismal prognosis, characterizing *RP11-137H2.4*sexact role and function in this process will be critical to the development of new therapeutic approaches to overcome GC resistance in children treated for cALL.

## INTRODUCTION

Childhood acute lymphoblastic leukemia (cALL) is the most common cancer among children under 14 years of age. Despite remarkable improvements in survival, with 5 year event-free survival rates of up to 85%, non-responding children with ALL still represent one of the most frequent cause of death from cancer in pediatrics.[1] Childhood ALL is a complex disease comprising multiple subtypes with distinctive somatic genetic alterations, including aneuploidy, chromosomal rearrangements, and point mutations.[1] These genetic alterations contribute to leukemogenesis by altering key regulatory processes, subverting normal proliferation control, blocking differentiation, and promoting resistance to death signals.[2] Despite this understanding of the molecular basis of this disease, accurate patient risk stratification is an ongoing challenge in cALL treatment and the development of innovative therapies. Several studies have described expression signatures for classifying molecularly-defined cALL subtypes and improving outcome prediction.[3–10] These studies focused on the analysis of protein-coding transcripts, probably because most of their translated proteins are important signaling molecules. A new class of non-coding RNAs, designated as long non-coding RNAs (lncRNAs) have been described recently. LncRNAs are expressed in most cell types and at most stages of development and play regulatory roles in various biological processes, including cell pluripotency and tumorigenesis.[11–15] LncRNAs can exert their effects through many cellular processes, such as spatial conformation of chromosomes, chromatin and DNA modifications, RNA transcription, pre-mRNA splicing, mRNA degradation, and mRNA translation.[13, 14] A well-known example of this is the *Hox* transcript antisense intergenic lncRNA, *HOTAIR*, which cooperates with the polycomb repressive complex to modulate gene expression. *HOTAIR* is deregulated in a spectrum of cancers, and its overexpression is associated with poor prognosis in breast, [16] liver, [17] colorectal, [18] gastrointestinal, [19] and pancreatic [20] cancers, and is proposed to increase tumor invasiveness and metastasis.[16] Recent expression studies performed on pre-B cALL samples have shown that lncRNA expression profiles can accurately classify disease subtypes and are correlated with outcome, [3, 21] (Lajoie *et al*., submitted). These studies suggest that lncRNAs might be utilized as diagnostic and prognostic markers in leukemia, but additional studies are needed to understand their clinical usefulness. Exploring the mechanisms underlying lncRNA functions is critical to recognizing their contribution to biological processes involved in cALL.[22]

In this report, we further investigated five lncRNAs that were shown to be specifically upregulated in pre-B cALLs (Lajoie et al. submitted; see Material and Methods for details). Silencing the expression of any one of these five lncRNAs in pre-B cALL cells had a significant impact on at least one of hallmark cancer traits (apoptosis, cell proliferation, migration, and treatment response). One of these lncRNAs, *RP11-137H2.4*, had a considerable impact on apoptosis, proliferation, and cell migration, or effectively restored glucocorticoid (GC) sensitivity in GC-resistant pre-B cALL cells. Glucocorticoids are the cornerstone of cALL therapy, inducing G1 arrest and apoptosis in leukemic blasts, and resistance to these agents significantly worsens prognosis. In addition, we showed that decreased expression of *RP11-137H2.4* modulates the NRAS/BRAF/NF-κB MAPK pathway that is then translated into a strong transcriptional modulation of E2F targets and NF-κB/Jun-Fos pathway members upon exposure to GCs. In summary, this study showed that the deregulation of a single lncRNA might interfere with apoptotic and GC responses. These observations strongly suggest that lncRNAs could have novel and unexplored therapeutic potential, at least in childhood pre-B ALL.

## RESULTS

### LncRNAs specifically overexpressed in pre-B cALL modulate cell proliferation and treatment response

**Figure 1:**
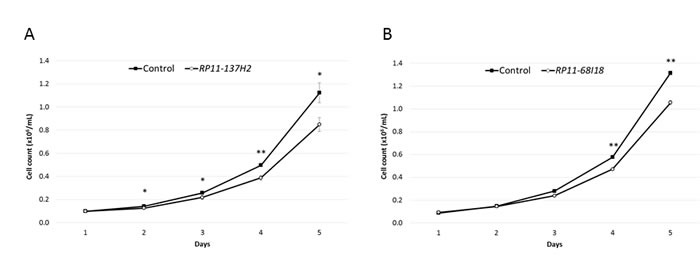
Silencing of lncRNAs upregulated in cALL reduces cell proliferation **A**. Reduction of cell proliferation in NALM-6 cells transfected with dsiRNA against *RP11-137H2.4*, measured by counting cells in triplicate each day for 5 days. **B**. Reduction of cell proliferation in NALM-6 cells transfected with siRNA against *RP11-68I18.10*, measured as in (A). Control cells were transfected with negative control siRNA/dsiRNA (see Methods). Comparisons were made using a two-tailed T-test. *: *P* ≤ 0.05; **: *P* ≤ 0.01.

**Figure 2:**
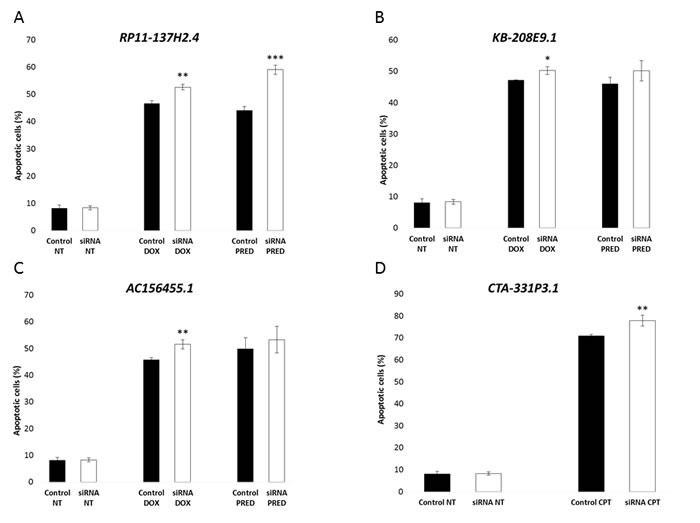
Silencing of lncRNAs upregulated in cALL increases apoptosis in response to cytotoxic treatment **A**. Increased apoptosis with doxorubicin and prednisolone treatment measured by AnnexinV / propidium iodide staining. NALM-6 cells transfected with dsiRNA against *RP11-137H2.4* were treated with 150 nM doxorubicin for 24 hours or with 750 µM prednisolone for 20 hours. DMSO was used as vehicle control for prednisolone. **B**. Increased apoptosis with doxorubicin and prednisolone treatment measured by AnnexinV / propidium iodide staining. NALM-6 cells transfected with dsiRNA against *KB-208E9.1* were treated with 150 nM doxorubicin for 24 hours or with 750 µM prednisolone for 20 hours. DMSO was used as vehicle control for prednisolone. **C**. Increased apoptosis with doxorubicin and prednisolone treatment measured by AnnexinV / propidium iodide staining. NALM-6 cells transfected with dsiRNAs against *AC156455.1* were treated with 150 nM doxorubicin for 24 hours or with 750 µM prednisolone for 20 hours. DMSO was used as vehicle control for prednisolone. **D**. Increased apoptosis with camptothecin treatment measured by AnnexinV / propidium iodide staining. NALM-6 cells transfected with siRNA against CTA-331P3.1 were treated with 500 nM camptothecin for 24 hours. Control cells were transfected with negative control siRNA/dsiRNA (see Methods). Comparisons were made using a two-tailed T-test. *: *P* ≤ 0.05; **: *P* ≤ 0.01; ***: *P* ≤ 0.001.

In a previous study, we identified 799 lncRNAs that were specifically overexpressed in primary pre-B cALL samples, as compared to CD10^+^CD19^+^ pre-B cells isolated from human cord blood (Lajoie et al. submitted; see Material and Methods above for details about cohort and gene expression analyses) (Figure S1). We validated that five such lncRNA transcripts overexpressed in pre-B cALL samples (*RP11-137H2.4*, *RP11-68I18.10, AC156455.1*, *KB-208E9.1*, and *CTA-331P3.1*) were also overexpressed in the Reh and NALM-6 pre-B cALL cell lines (Figure S2). We then assessed their impact on cancer traits, such as cell proliferation and apoptosis, through siRNA-mediated loss-of-function assays in NALM-6 (average silencing of 61.4%; see Figure S3). Two lncRNAs, *RP11-137H2.4* and *RP11-68I18.10*, caused a significant reduction in proliferation (20-24%, *P* ≤ 0.05; see Figure 1) when silenced, while knockdown of *AC156455.1*, *KB-208E9.1*, or *CTA-331P3.1* had no effect on proliferation (data not shown). Furthermore, *RP11-137H2.4*, *AC156455.1*, *KB-208E9.1*, or *CTA-331P3.1* knockdown significantly increased apoptosis (3-15%, *P* ≤ 0.05) when treated with either camptothecin (CPT), doxorubicin (DOX), or prednisolone (Figure 2), while silencing *RP11-68I18.10* had no effect on apoptosis (data not shown). Although the magnitudes of the effects on apoptosis are relatively modest (yet statistically significant), they are the product of the deregulation of single lncRNAs in pre-B cALL. These results thus show that individual lncRNAs can have large impacts on cancer phenotypes.

**Figure 3:**
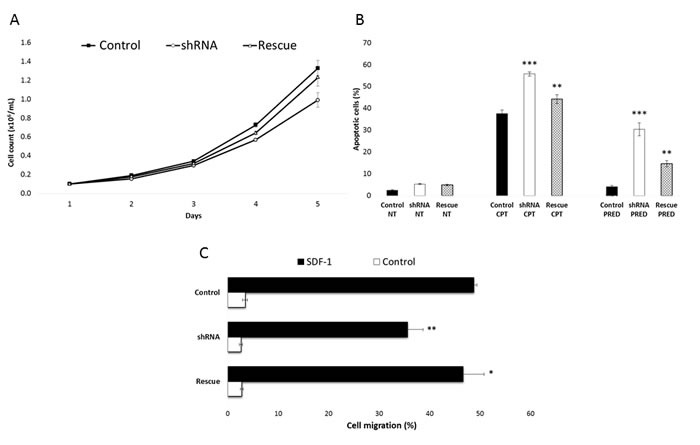
***RP11-137H2.4*** silencing restores normal glucocorticoid apoptotic response. **A**. Reduction of cell proliferation in Reh cells stably transduced with shRNAs against *RP11-137H2.4* (“shRNA”) and partial rescue of the phenotype by overexpressing the lncRNA in a cell line where *RP11-137H2.4* is silenced (“Rescue”, see Methods), measured by counting cells in triplicate each day for 5 days. **B**. Increased apoptosis with camptothecin or prednisolone treatment and partial rescue of the phenotype by overexpressing the lncRNA measured by AnnexinV / propidium iodide staining. Reh cells stably transduced with shRNAs against *RP11-137H2.4* were treated with 10 µM camptothecin for 6 hours or 750 µM prednisolone for 24 hours. DMSO was used as vehicle control for prednisolone. **C**. Reduction of cell migration in Reh cells stably transduced with shRNAs against *RP11-137H2.4* and partial rescue of the phenotype by overexpressing the lncRNA, measured by counting cells in triplicate, using SDF-1 as a chemoattractant. Control cells were stably transduced with scrambled shRNA. Comparisons were made using a two-tailed T-test. *: *P* ≤ 0.05; **: *P* ≤ 0.01; ***: *P* ≤ 0.001.

### *RP11-137H2.4* knockdown inhibits cell proliferation and migration and restores glucocorticoid sensitivity

The lncRNA *RP11-137H2.4* had the most pronounced impact upon siRNA-mediated silencing in NALM-6 cells, with an observed 24% reduction of proliferation at 5 days (Figure 1A) and increased apoptosis by 6% and 15% after exposure to DOX or prednisolone, respectively (Figure 2A). We repeated these experiments in a second pre-B cALL cell line, Reh that overexpresses this lncRNA (Figure S2a). Since, contrary to NALM-6, the Reh cell line is hard to transfect we stably transduced Reh with a shRNA specifically targeting *RP11-137H2.4*. We then validated the silencing (Figure S4) and repeated the phenotyping analysis (Figure 3). The sensitivity of this stable cell line was exacerbated for CPT and prednisolone, with 18% and 26% cell death after treatment, respectively (Figure 3B). This is particularly interesting since Reh does not express functional NR3C1 GC receptor (GR) protein (confirmed in our lab, data not shown) and is therefore resistant to GCs.[23] Furthermore, a significant decrease (27%; *P* ≤ 0.005) in cell migration was also observed (Figure 3C). Since *RP11-137H2.4* has only one documented isoform (Figure S5), rescue experiments performed by introducing a vector constitutively overexpressing *RP11-137H2.4* in cells expressing this shRNA (Figure S4) could be performed. These resulted in increased cell proliferation, partial rescue of CPT- and prednisolone-induced apoptosis, and partial rescue of migration, indicating that these effects were specific to *RP11-137H2.4* silencing (Figure 3). These results strongly suggest that *RP11-137H2.4* silencing specifically restores GC sensitivity in a NR3C1-independent manner in the Reh and, possibly, NALM-6 cell lines.

**Figure 4:**
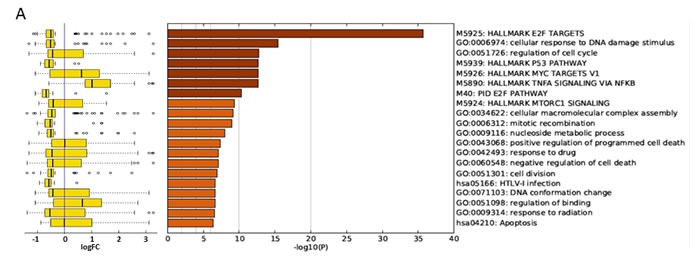

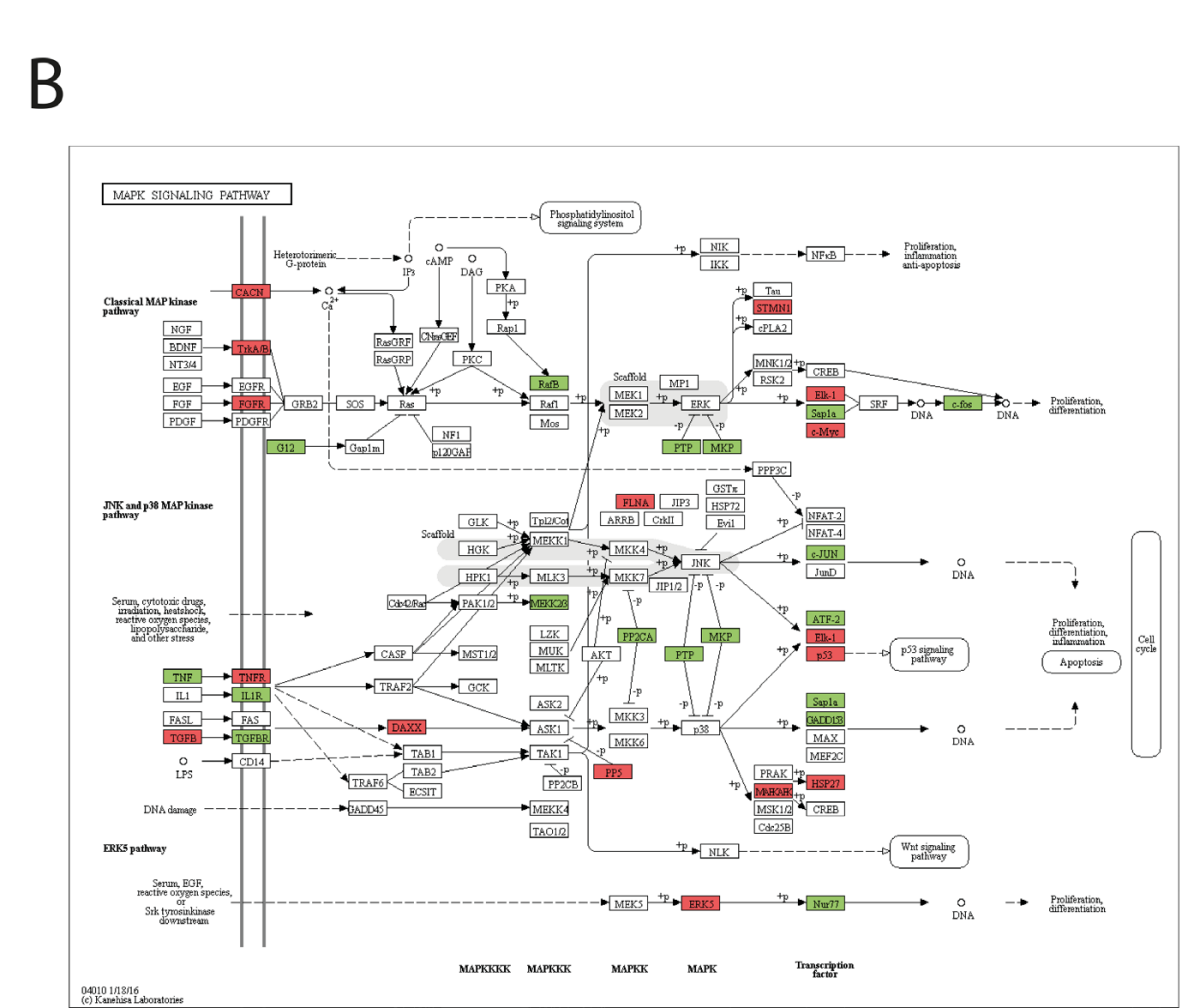

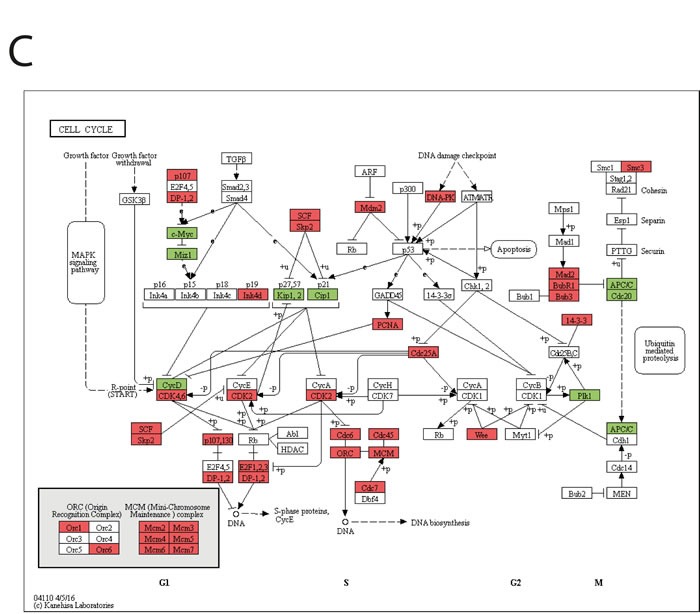
Gene ontology (GO) analysis of ***RP11-137H2*** silencing-specific gene expression deregulation upon prednisolone exposure. Whole-transcriptome deep sequencing was performed on Reh cell lines expressing either scrambled or *RP11-137H2.4*-specific shRNA. Cells were exposed for 24 h to either vehicle (DMSO) or 750 µM prednisolone. **A**. Deregulated genes’ enriched GO categories are reported with significance (-log10(P)) (right) along with a box-plot of log2 expression fold changes for the corresponding GO categories (left). Edges of the box are the first and third quartiles, while the band inside the box is the median. Whiskers represent minimum and maximum values. **B**. Genes in the MAPK cascade deregulated in Reh cells following *RP11-137H2.4* silencing and prednisolone treatment (red is downregulated, green is upregulated). **C**. Genes in the cell cycle pathway deregulated in Reh cells following *RP11-137H2.4* silencing and prednisolone treatment (red is downregulated, green is upregulated).

### *RP11-137H2.4* silencing modulates the MAPK and cell cycle pathways to restore prednisolone sensitivity

In light of these results, we further investigated how *RP11-137H2.4* silencing affected global transcriptional profiles upon prednisolone exposure. We first identified genes significantly deregulated *RP11-137H2.4* silencing upon prednisolone exposure by sequencing the whole transcriptome of cells expressing *RP11-137H2.4* shRNA exposed to prednisolone and comparing it to cells expressing *RP11-137H2.4* shRNA exposed to vehicle (DMSO). Gene ontology (GO) analyses indicated that E2F target genes, DNA damage response, and p53 pathway and TNFα/NF-κB signaling genes were significantly deregulated by *RP11-137H2.4* silencing upon prednisolone exposure (Figure 4A, right). Interestingly, genes in cell cycle-related categories were specifically downregulated by *RP11-137H2.4* silencing, while those corresponding to TNFα/NF-κB signaling were upregulated (Figure 4A, left). Closer examination of the cell cycle and MAPK extracellular signal transduction cascade pathways confirmed that they were indeed down- and up-regulated, respectively, by *RP11-137H2.4* silencing upon prednisolone exposure (Figure 4B-4C). Furthermore, we observed a significant overlap (*p* ≤ 0.005, exact hypergeometric probability) between genes significantly deregulated by *RP11-137H2.4* silencing upon prednisolone exposure in our dataset (1131 transcripts at FDR ≤ 0.005) and GC-induced genes whose promoters contain glucocorticoid-response elements (GREs) (Reddy *et al*.) (Table 1).[24] Indeed, 10/61 of the genes in the latter list were differentially expressed in our experiments and were consistently expressed in the same direction (up- or down-regulated) in both datasets, with the exception of *CKB*, suggesting that GC response genes are modulated specifically by *RP11-137H2.4* silencing upon prednisolone exposure. These data strongly suggest that *RP11-137H2.4* expression in pre-B cALL can abrogate normal GC response by modulating the expression of members of MAPK cascade and cell cycle pathways in a NR3C1-independent fashion.

Interestingly, *RP11-137H2.4* silencing in vehicle-treated (DMSO) conditions, as compared with control cells expressing a scrambled shRNA, also seemed to significantly affect gene expression profiles (Figure S6). Indeed, GO analyses revealed significant enrichment of deregulated genes in several GO categories related to external signal transduction, cell differentiation, adhesion, and morphogenesis (Figure S5a, right). Although no clear overall direction was observed in gene expression modulation for these GO categories (Figure S5a, left), the expression of multiple key members of the MAPK extracellular signal transduction cascade and cell cycle pathways was significantly altered (Figure S5b-c).

Taken together, these data indicate that pre-B cALL-specific lncRNA overexpression can induce malignant behaviors, such as increased cell proliferation, resistance to apoptosis, and increased cell migration. Finally, our best candidate, *RP11-137H2.4*, seems to be implicated in GC resistance in pre-B cALL patients.

**Table 1:** GC-modulated genes common to both Reddy et al. and our datasets

Gene Name	Ensembl ID	Log2 Fold-change (Reddy et al.)	Log2 Fold-change (Ouimet et al.)	FDR(Ouimet et al.)
ZFP36	ENSG00000128016	2.29	0.99	1.95E-06
BIRC3	ENSG00000023445	1.87	2.48	8.54E-11
ERN1	ENSG00000178607	1.11	0.46	3.53E-03
ALOX5AP	ENSG00000132965	1.02	0.74	2.58E-03
CEBPB	ENSG00000172216	0.98	0.59	1.57E-03
FOSL2	ENSG00000075426	0.80	0.66	3.79E-03
CKB	ENSG00000166165	0.71	−0.69	1.52E-04
RHOB	ENSG00000143878	0.46	1.24	2.72E-16
KLF5	ENSG00000102554	0.44	1.23	1.21E-03
ID3	ENSG00000117318	−0.46	−0.44	2.37E-05

## DISCUSSION

To date, childhood leukemia research has mainly focused on the deregulated expression of protein-coding genes that could be used as diagnostic and prognostic biomarkers. In this study, we have shown that five lncRNAs specifically overexpressed in pre-B cALL can significantly impact cancer traits, such as cell proliferation and migration, apoptosis, and treatment response. Furthermore, while the magnitude of the observed effects was relatively modest (while still statistically significant), they are caused by the partial silencing of single lncRNAs. The importance of this cannot be understated: modulating the expression of individual, non-coding transcripts can significantly impact cancer hallmark phenotypes. Moreover, we demonstrated that *RP11-137H2.4* expression in cALL is linked to GC treatment resistance, and that its silencing is sufficient to restore a NR3C1-independent cellular response to these compounds, leading to GC-induced apoptosis. Furthermore, stably overexpressing *RP11-137H2.4* in Reh cells already expressing the shRNA specific to it resulted in a partial rescue of these phenotypes, further demonstrating the *RP11-137H2.4* specificity of the effects we observed.

Deregulation of lncRNAs has been linked to several complex human diseases, including cancer.[15, 25] For instance, *MALAT1* was found to be highly expressed and associated with metastasis and poor prognosis in many cancer types [26] including non-small cell lung carcinoma [27, 28] and hepatocellular carcinoma.[29] The up-regulation of several other lncRNAs, such as *HOTAIR* and *MVIH*, and the downregulation of *H19* have been associated with poor prognosis in cancers.[30] The up-regulation of lncRNA *PCA3* (*DD3*) has proven to be a reliable biomarker for early detection of prostate cancer.[31] To date, few lncRNAs have been directly linked to leukemogenesis. In childhood pre-B ALL, two studies showed that expression of lncRNAs correlated with cytogenetic abnormalities, disease subtypes, and survival of B-ALL patients.[21] (Lajoie et al. submitted) The B-ALL-associated long RNA-2 (*BALR-2*) has been shown to be specifically upregulated in MLL-rearranged ALL.[21] *BALR-2* was identified as a modulator of the response to GC treatment.[21]

These results are very interesting considering that GCs are key chemotherapeutic agents in pre-B cALL therapy. GCs are involved in many biological processes, such as metabolism, development, differentiation, immunity, reproduction, and neural activity. The diverse actions of GC have led to their use as therapeutic agents in the treatment of many diseases, including cancer. Their effect on lymphoid cells is dramatic, and includes the induction of G1 cell cycle arrest and apoptosis.[32] Furthermore, relapsed cALL patients acquire prednisolone resistance disproportional to other anti-leukemic agents. Indeed, resistance to GCs is a hallmark of relapsed cALL, since relapsed leukemia blasts are significantly more resistant to prednisolone and dexamethasone, and is a strong predictor of negative outcome at diagnosis.[32–35]

Several members of key signalling pathways were overrepresented in the transcripts deregulated by *RP11-137H2.4* silencing. Indeed, we found that *NRAS*, *BRAF*, *NF-κB*, and several other genes in the MAPK cascade were downregulated following *RP11-137H2.4* silencing, whereas members of the activating protein-1 (AP-1) complex, *JUN* and *FOS*, were upregulated. Genes involved in cell cycle control were also affected. Prednisolone treatment showed a strong, *RP11-137H2.4* silencing-specific repression of E2F target genes involved in cell cycle control and a modulation of MAPK cascade gene expression, as expected in GC-sensitive cells. Importantly, *RP11-137H2.4* silencing restores GC sensitivity in the Reh prednisolone-resistant pre-B cALL cell line, despite it having both copies of the *NR3C1* GC receptor gene inactivated (one allele is deleted while the other carries a nonsense mutation (p.Gln528*), which we confirmed independently; data not shown).[36] Prednisolone, a GC, diffuses passively into the cell, where it binds the GC receptor (NR3C1, also known as GR), causing it to dimerize. Dimerized GR acts as a transcription factor by either binding to positive or negative glucocorticoid response elements in the DNA, or by binding to other transcription factors, such as NF-kB and AP-1.[37] The AP-1 family of transcription factors consists of multiple Jun and Fos members and integrates growth signals at the transcriptional level. AP-1 factors regulate the expression of *CCND1* and *E2F*, which in turn regulates E2F-downstream genes, leading to the modulation of cellular proliferation, differentiation, apoptosis, oncogene-induced transformation, and cancer cell invasion.[38] While further studies are required to characterize the molecular mechanisms involved, our results strongly suggest that *RP11-137H2.4* expression abrogates normal GC response in pre-B cALL samples by modulating the expression of MAPK cascade genes. These results are strikingly similar to those of Fernando *et al*. where they demonstrated that knocking down another lncRNA specifically overexpressed in pre-B cALL, *BALR-2*. They found that knockdown of *BALR-2* restored prednisolone sensitivity through modulation of the GC receptor signaling pathway, specifically by upregulating *JUN*, *FOS*, *SGK1*, and *SERPINE1*. We also see these genes upregulated by prednisolone, but not DMSO, in our system (Tables S2 and S3, respectively).[21]

In summary, we found that specific lncRNAs play important roles in pre-B cALL progression and treatment response. In particular, one lncRNA, *RP11-137H2.4,* seems to play a role into GC resistance in treatment of cALL. Characterizing its exact role and function in this process will be critical to the development of new therapeutic approaches to overcome GC resistance in children treated for cALL.

## MATERIALS AND METHODS

### Childhood ALL sample cohort and transcriptome profiling

Our study cohort consisted of 56 pre-B cALL patients (28 females and 28 males) with a mean age at diagnosis of 6.1±3.6 years. All subjects were French-Canadians of European descent diagnosed in the Division of Hematology-Oncology at the Sainte-Justine Hospital (Montreal, Canada) and part of the Quebec childhood ALL cohort (QcALL).[39] CD10^+^CD19^+^ cells isolated from human cord blood were used as controls. Briefly, after being isolated using a Ficoll-Paque gradient fragmentation, PBMCs were positively selected using MACS Separation with CD19 MicroBeads (Miltenyi Biotec). Cell sorting was performed on the CD19^+^ cells using CD19-PE and CD10-FITC antibodies (Miltenyi Biotec). Purity was >90 %. Total RNA was extracted from white blood cell pellets obtained from bone marrow and peripheral blood at diagnosis using the mirVana Isolation kit (Ambion) according to manufacturer's protocol. Following a DNAse treatment to remove possible contamination by genomic DNA, ribosomal RNAs were removed using the RiboMinus Eukaryote kit (Invitrogen). cDNA libraries were prepared using the SOLiD Total RNA-seq kit based on manufacturer's protocol and sequenced on the Life Technologies SOLiD 4/5500 System (paired-end: 50×35bp and 75×35bp). Reads were aligned to the human genome (hg19) using the Lifescope Genomic Analysis Software (Applied Biosystems; Whole Transcriptome Analysis pipeline, default parameters). Expression levels by gene were determined with the HTseq-count software[40] using gene models from Ensembl75 combined with (non-overlapping) lncRNA transcripts provided in Casero et al.[41] The identification of differentially expressed transcripts relative to HCB controls was done using the Generalized Linear Model implemented in the edgeR package.[42] The Sainte-Justine Institutional Review Board approved the research protocol, and informed consent was obtained from all participating individuals and/or their parents.

### Cell culture

Reh (Human B cell precursor leukemia; ATCC CRL-1567) and NALM-6 (Human B cell precursor leukemia; DSMZ ACC-128) cells were cultured in RPMI 1640 medium (Wisent) supplemented with 10% heat-inactivated fetal bovine serum (FBS) (Wisent). Reh_shRNA control and Reh_shRNA-*RP11-137H2.4* cells were cultured in RPMI 1640 medium (Wisent) supplemented with 10% heat-inactivated FBS and 1µg/mL puromycin (Wisent). Reh_shRNA-*RP11-137H2.4*_*RP11-137H2.4* cells (“Rescue” cell line) were cultured in the same media as Reh_shRNA *RP11-137H2.4*, but supplemented with 5µg/mL blasticidin (Wisent). Human embryonic kidney 293T cells (HEK293T; ATCC CRL-3216) were cultured in Dulbecco's modified Eagle's medium (DMEM) (Wisent) supplemented with 10% heat-inactivated FBS. All culture media were supplemented with 1% penicillin/streptomycin (Wisent), and all cell lines were cultured in a 37 °C incubator.

### RT-qPCR

Total RNA was extracted from cells using RNeasy mini Kit (Qiagen). Total RNA was retro-transcribed into cDNA using the QuantiTect Reverse Transcription Kit (Qiagen), and qPCR amplifications (triplicates) were performed on the ABI Prism 7000 Sequence Detection System (Thermo Fisher Scientific) using SYBR Green PCR Master Mix (Applied Biosystems). Primer sequences used are listed in Table S1. The cycling parameters were 95 °C for 10 min, 40 cycles (95°C for 15 sec, 60°C for 1 min) followed by a denaturation curve at 60°C. GAPDH was used as reference gene. Expression values were calculated as 2^-(∆∆CT)^, as per Livak and Schmittgen.[43]

### siRNA-mediated silencing

Custom Silencer Select siRNA (Ambion by Life Technologies) and Custom dsiRNA Duplex (Integrated DNA Technologies) were used to silence the lncRNA. Silencer Select negative control No.1 siRNA (Ambion) and dsiRNA NC1 (Integrated DNA Technologies) were used as negative control siRNA/dsiRNA. Lonza's Nucleofector™ Technology was used for the transfection of siRNA/dsiRNA into the NALM-6 cells using the Nucleofector kit T (Lonza) and the program C-005; silencing was measured 24 hours later by RT-qPCR.

### shRNA and overexpression constructs

The structure region of *RP11-137H2.4* (Ensembl ENSG00000226659) was used for cDNA synthesis (IDT), which was subcloned into the pLenti vector with blasticidin resistance using Gateway technology. The integrity of the clone selected was confirmed by sequencing. shRNA target sequences for *RP11-137H2.4* were cloned into the psi-LVRU6P vector with puromycin resistance (GeneCopoeia). A scrambled shRNA for psi-LVRU6P (GeneCopoeia) was used as control.

### Vector particle production and transduction

For preparation of lentiviral particles, we used a co-transfection procedure. Briefly, 293T cells were co-transfected with 12 µg of the pREV vector, 15.6 µg of the pVSVG vector, 30 µg of the pMDL vector, and 18 µg of the pLenti vector containing the gene of interest or the shRNA of the gene of interest using Lipofectamine 2000 (Invitrogen) to produce lentiviral particles. Forty-eight hours later, vector particles were collected from culture supernatants, filtered through 0.22 µm pore-size nitrocellulose membranes, concentrated, and aliquoted before being frozen at -80 °C until use. The amount of viral particles produced was determined by the HIV-1 p24 antigen capture assay (ABL Inc.). Transductions of Reh cells were performed using 1 × 10^6^ cells and 600 ng of lentiviral particles in the presence of 8 µg/mL polybrene (Sigma-Aldrich). Forty-eight hours after infection, cells were placed in medium containing antibiotics for selection.

### Proliferation, apoptosis, and drug response assays

To measure proliferation, 150µL of cells at 0.1 × 10^6^ cells/mL were plated in 96-well plates in triplicate for five days. Each day, cells were counted in triplicate using a cell counter (Beckman Coulter). For drug response assays, 2mL of cells were diluted to 0.5 × 10^5^ cells/mL in 12-well plates in their culture medium and treated with the corresponding agent, camptothecin (Tocris Bioscience), doxorubicin (Sigma-Aldrich), prednisolone (Sigma-Aldrich) to promote DNA damage-induced apoptosis, then harvested to assay for apoptosis. DMSO (vehicle) was used as control for prednisolone treatment, while other drugs were diluted in water. Cell apoptosis was measured using the Alexa Fluor® 488 Annexin V/Dead Cell Apoptosis Kit (Thermo Fisher Scientific) according to the manufacturer's instructions. Stained cells were analyzed by flow cytometry using the BD FACSCanto^TM^ II cell analyser and the BD FACSDiva software (BD Biosciences) according to manufacturer's guidelines.

### Cell migration assay

To measure migration, 30 µL of media containing 30 ng/mL of the chemoattractant SDF-1 (ProSpec) or not (controls) were filled in triplicate into microplate wells of a 96-well cell migration system (ChemoTx #101-5) with 5 µm pore size (Neuro Probe). 25µL of cells at 8 × 10^6^ cells/mL were dropped on the filter of each well, and the filled instrument was incubated at 37 °C in humidified air with 5% CO_2_ for 1.5 hours. After incubation, 25µL of migrated cells in the microplate were counted using a cell counter (Beckman Coulter).

### Whole transcriptome sequencing

Total RNA was extracted from white blood cell pellets obtained from bone marrow and peripheral blood at diagnosis using the mirVana Isolation kit (Ambion) according to the manufacturer's protocol. Following a DNase treatment to remove possible contamination by genomic DNA, ribosomal RNAs were removed using the RiboMinus Eukaryote kit (Invitrogen). cDNA libraries were prepared using the Illumina TruSeq Stranded Total RNA Library Prep Kit based on manufacturer's protocol and sequenced on an Illumina HiSeq 2500 (2 × 100 bp paired-end).

Sequencing data were analyzed using the GATK best practice pipeline.[44] Expression levels by gene were determined with the HTseq-count software[40] using gene models from Ensembl75 combined with (non-overlapping) lncRNA transcripts provided in Casero et al.[41] Transcript differential expression was assessed using Deseq2.[45]

### Gene expression and ontology analyses

Differential gene expression analyses were performed using Deseq2.[45] First, genes regulated by the shRNA in DMSO were retrieved using the design “*~ group*”, where “group” corresponds to the presence/absence of the shRNA or scrambled control. Positive gene expression log_2_ fold-changes in that design correspond to genes being overexpressed in the presence of the shRNA in DMSO. Second, genes having different expression log_2_ fold-changes in prednisolone vs. DMSO due to the presence of the shRNA were retrieved using the design “*~ treatment + shRNA + treatment:shRNA*”, where “treatment” corresponds to prednisolone/DMSO and “shRNA” corresponds to presence of the shRNA or scrambled control. Positive gene expression log_2_ fold-changes in that design correspond to genes having a positive shRNA-specific log_2_ fold-change difference in prednisolone vs. DMSO. The top 500 differentially expressed genes (p-adjusted ≤ 0.05) of the above two designs were used as input into Metascape (http://metascape.org/; Aug 2016) for the Gene Ontology (GO) analyses. Boxplots of gene expression log_2_ fold-changes were generated based on genes assigned to the enriched GO terms. Significantly differentially expressed genes were mapped on the KEGG MAPK signaling and cell cycle pathways.[46]

## SUPPLEMENTARY MATERIALS TABLES FIGURES





## Abbreviations

cALL: childhood acute lymphoblastic leukemia
lncRNA: long non-coding RNA
GC: glucocorticoid
siRNA / dsiRNA: short interfering RNA / double-stranded short interfering RNA
shRNA: short hairpin RNA
CPT: camptothecin
DOX: doxorubicin
GO: gene ontology
QcALL: Quebec childhood ALL cohort
RT-qPCR: quantitative reverse transcription polymerase chain reaction
