# Supplementary material for: A childhood acute lymphoblastic leukemia-specific lncRNA implicated in prednisolone resistance, cell proliferation, and migration
Source: Oncotarget. 2016 Dec 15;8(5):7477–88. doi: 10.18632/oncotarget.13936 (PMC5352336; doi:10.18632/oncotarget.13936)
Supplement: Supplementary file 1 [file oncotarget-08-7477-s001.pdf]

A childhood acute lymphoblastic leukemia-specific lncRNA implicated in prednisolone resistance, cell proliferation, and migration

Supplementary Material

Figure S1

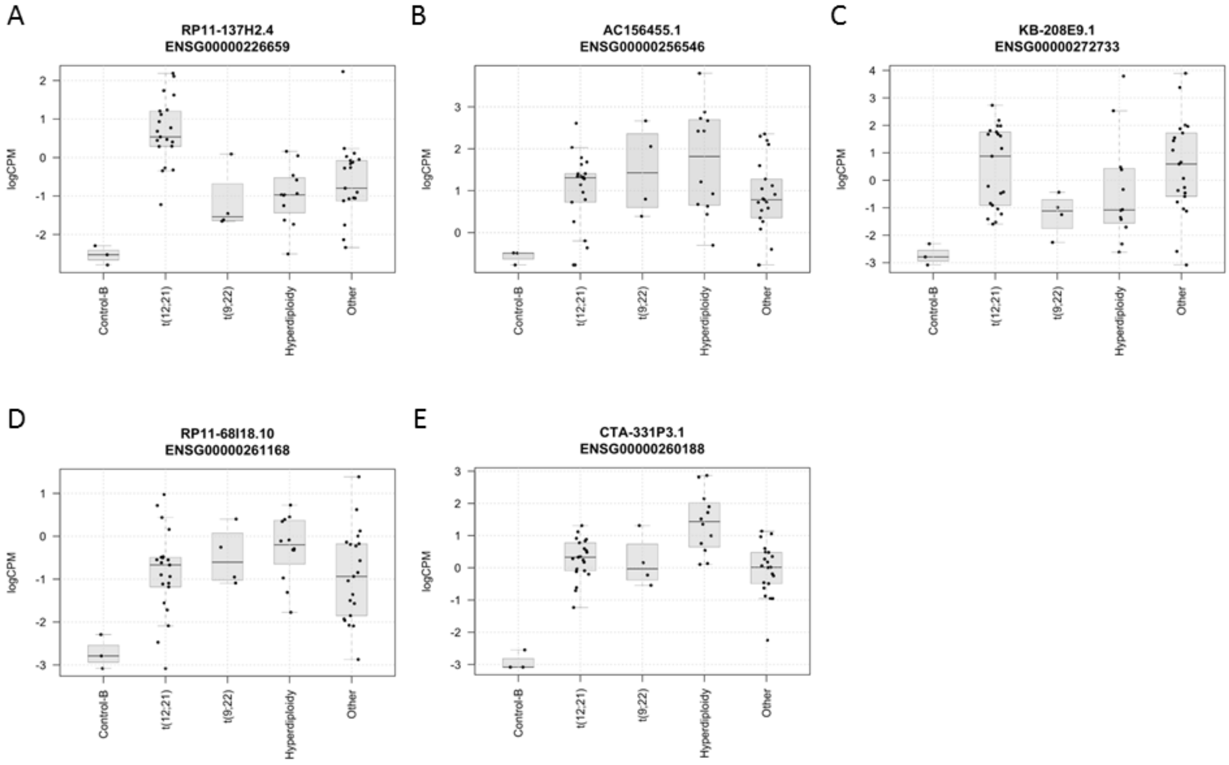

Figure S1. Overexpression of five lncRNAs in pre-B cALL patients. Box-plots representing lncRNA expression in a cohort of 56 cALL patients divided by subtype. Fold-changes obtained by comparison to normal CD19$^+$ cells obtained from human cord blood. (A) *RP11-137H2.4*; (B) *AC156455.1*; (C) *KB-208E9.1*; (D) *RP11-68I18.10*; (E) *CTA-331P3.1*. Edges of the box are the first and third quartiles, while the band inside the box is the median. Whiskers represent minimum and maximum values.

Figure S2

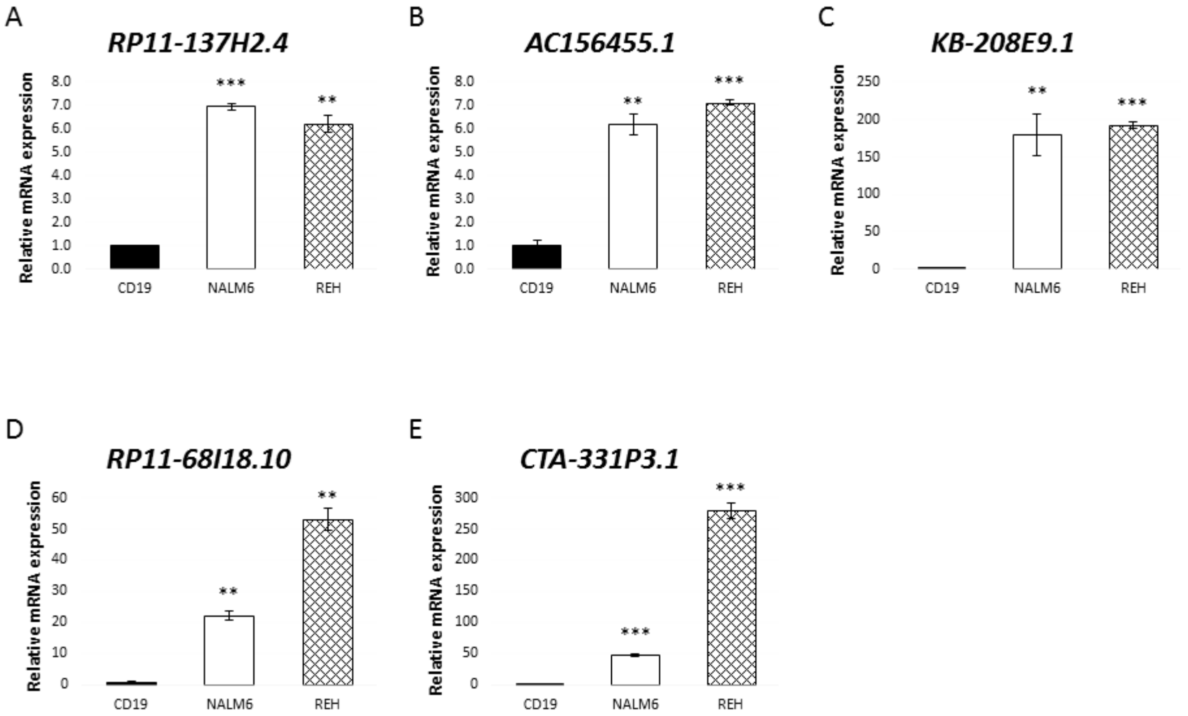

Figure S2. Validation of lncRNA upregulation in the Reh and NALM-6 pre-B cALL cell lines. LncRNA expression was assessed by RT-qPCR relative to that of normal $CD19^+$ cells obtained from human cord blood. Expression was normalized to *GAPDH*. (A) *RP11-137H2.4*; (B) *AC156455.1*; (C) *KB-208E9.1*; (D) *RP11-68I18.10*; (E) *CTA-331P3.1***.**

Figure S3

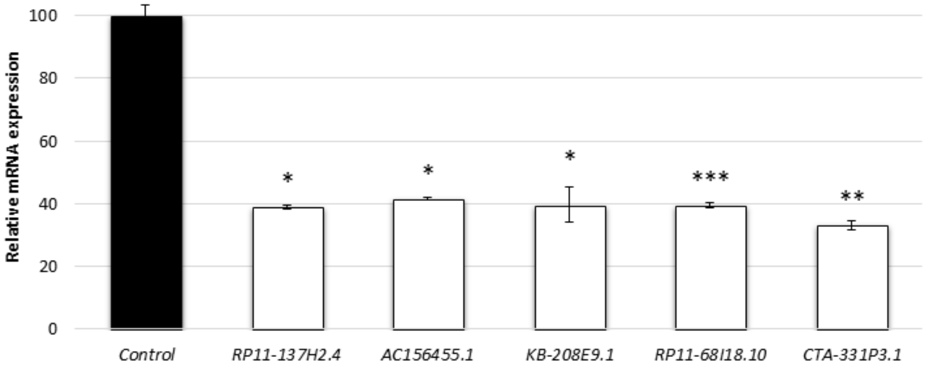

Figure S3. siRNA-mediated silencing of five lncRNAs in NALM-6 cells. siRNA-mediated knockdown of 5 lncRNAs in NALM-6 cells: *RP11-137H2.4*, *AC156455.1*, *KB-208E9.1*, *RP11-68I18.10* and *CTA-331P3.1*, as measured by RT-qPCR (normalized to GAPDH). siRNAs against each lncRNA were introduced in NALM-6 cells by nucleofection, and the expression of these lncRNAs was measured after 24 hours. Control were transfected with scrambled siRNA.

Figure S4

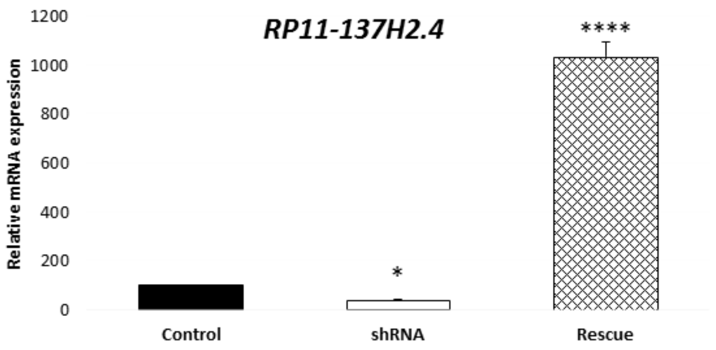

Figure S4. Expression of the *RP11-137H2.4* lncRNA after shRNA-mediated silencing and rescue. *RP11-137H2.4* expression in a stably-transfected Reh cell line expressing shRNA specific to *RP11-137H2.4* ("shRNA") or a stably-transfected Reh cell line expressing both an shRNA specific to *RP11-137H2.4* and a vector overexpressing *RP11-137H2.4* ("Rescue"), as measured by RT-qPCR (normalized to GAPDH). Comparisons were made using a two-tailed T-test. *: P≤0.05.

Figure S5

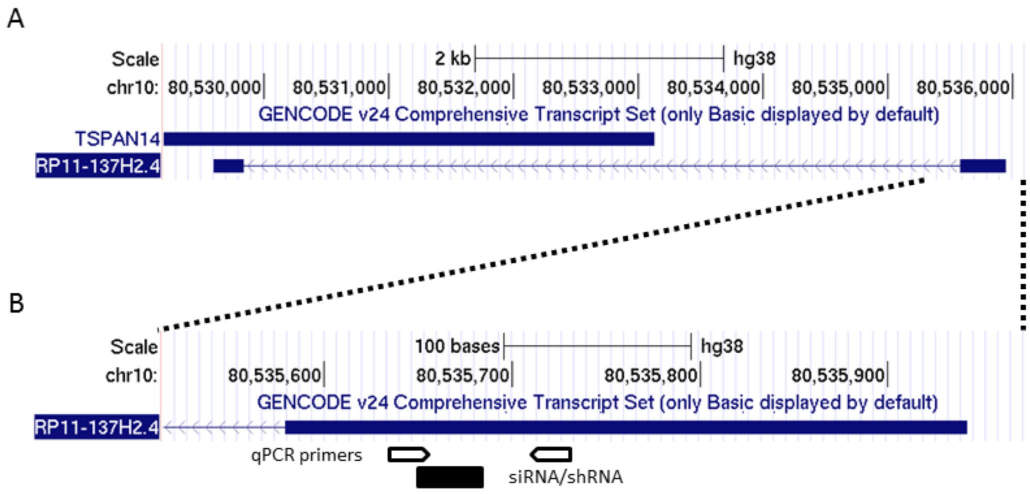

Figure S5. *RP11-137H2.4* genomic structure. (A) Genomic structure of *RP11-137H2.4*. (B) Zoom in on the 1st exon of *RP11-137H2.4*. qPCR primers' and siRNA/shRNA locations are indicated. Images were generated using the University of California Santa Cruz' (UCSC) Genome Browser, human genome build hg38 (https://genome.ucsc.edu/)

Figure S6

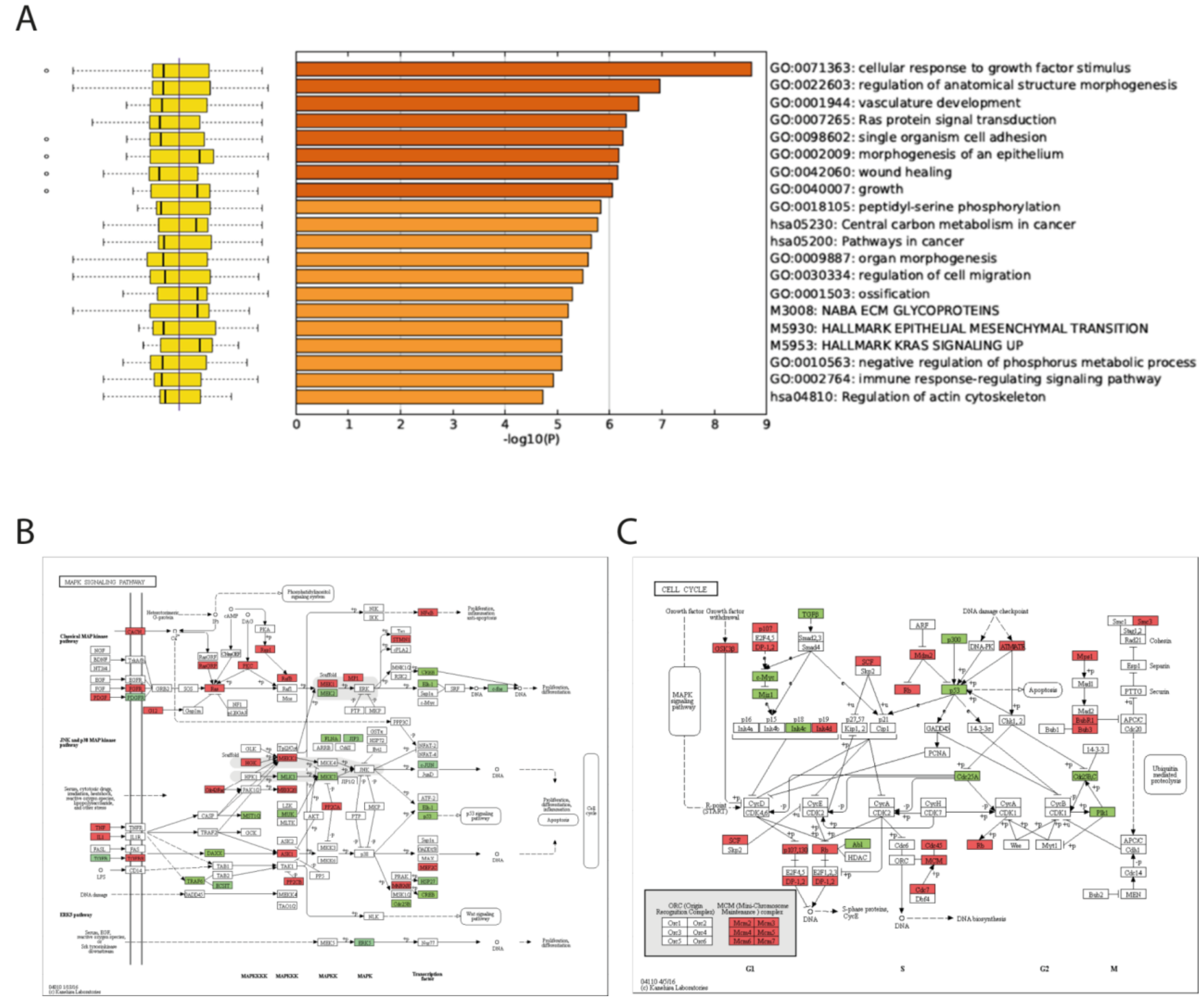

Figure S6. Gene ontology (GO) analysis of *RP11-137H2.4* silencing-specific gene expression deregulation upon DMSO exposure. Whole-transcriptome deep sequencing was performed on Reh cell lines expressing either scrambled or *RP11-137H2.4*-specific shRNA exposed for 24 h to DMSO. (A) Deregulated genes' enriched GO categories are reported with significance (-log10(P)) (right) along with a box-plot of log2 expression fold changes for the corresponding GO

categories (left). Edges of the box are the first and third quartiles, while the band inside the box is the median. Whiskers represent minimum and maximum values. (B) Genes in the MAPK cascade deregulated in Reh cells following *RP11-137H2.4* silencing and DMSO treatment (red is downregulated, green is upregulated). (C) Genes in the cell cycle pathway deregulated in Reh cells following *RP11-137H2.4* silencing and DMSO treatment (red is downregulated, green is upregulated).
