# Supplementary material for: A childhood acute lymphoblastic leukemia-specific lncRNA implicated in prednisolone resistance, cell proliferation, and migration
Source: Oncotarget. 2016 Dec 15;8(5):7477–88. doi: 10.18632/oncotarget.13936 (PMC5352336; doi:10.18632/oncotarget.13936)
Supplement: Supplementary file 2 [file oncotarget-08-7477-s002.docx]

**Table S1. Primers and siRNAs sequences.**

| **siRNA sequences** | | | |
| --- | --- | --- | --- |
| **Type** | **Manufacturer** | **Gene** | **Sequence** |
| dsiRNA | IDT | RP11-137H2.4 | 5'-CUUCAUUGUGACAGGAGUAGAGGUCCA-3' |
| dsiRNA | IDT | AC156455.1 | 5'-AGCAUCAUAUGUUGGAUGAUUGCAGUU-3' |
| dsiRNA | IDT | AC156455.1 | 5'-ACAGAAGAGGAUGCCUCAUUGGUCCAA-3' |
| dsiRNA | IDT | KB-208E9.1 | 5'-CGAUGGAAGAUCUACUUCUUGCUGGAA-3' |
| siRNA | Ambion | RP11-68I18.10 | 5'-GGUUUAAAUAUAGACCUAAtt-3' |
| siRNA | Ambion | CTA-331P3.1 | 5'-GCACAAUCCUAUCUAGACAtt-3' |
| shRNA | Genecopoeia | RP11-137H2.4 | 5'-ACCTCTACAGGGAGCAGATTA-3' |

| **RT-qPCR primers** | | | |
| --- | --- | --- | --- |
| **Gene** | **Manufacturer** | **Primer name** | **Sequence** |
| RP11-137H2.4 | IDT | RP11-137H2.4_RT_F | 5'-TGCAAGTGACTGGAAACCAC-3' |
| RP11-137H2.4 | IDT | RP11-137H2.4_RT_R | 5'- CGACGAAACAGTCGACTTCA-3' |
| AC156455.1 | IDT | AC156455.1_RT_F | 5'-GGGTCTTACCCGACGTGAG-3' |
| AC156455.1 | IDT | AC156455.1_RT_R | 5'-CGCGTGTTGAGGACTACAGA-3' |
| KB-208E9.1 | IDT | KB-208E9.1_RT_F | 5'-TGCATTATCTCCCAACACCA-3' |
| KB-208E9.1 | IDT | KB-208E9.1_RT_R | 5'-AGGTTCCTCTCCTTCCTCCA-3' |
| RP11-68I18.10 | IDT | RP11-68I18.10_RT_F | 5'-GCGAAGGCATGGTATCTTGT-3' |
| RP11-68I18.10 | IDT | RP11-68I18.10_RT_R | 5'-AAAGGGAAAGGGATTGCCTA-3' |
| CTA-331P3.1 | IDT | CTA-331P3.1_RT_F | 5'-CCAGAGCAGAGCATCAAGAG-3' |
| CTA-331P3.1 | IDT | CTA-331P3.1_RT_R | 5'-GAAGCACTTTGGGAAATGGA-3' |

**Table S2. Top-100 *RP11-137H2.4* silencing-specific deregulated genes upon 24h prednisolone treatment.**

| **Gene symbol** | **Gene name** | **Log2 Fold-Change** | **Log2 Fold-change SE** | **P-value** | **Adjusted P-value** |
| --- | --- | --- | --- | --- | --- |
| PLK2 | ENSG00000145632 | 2.16 | 0.16 | 8.83E-40 | 1.23E-35 |
| FOS | ENSG00000170345 | 3.11 | 0.25 | 3.91E-37 | 2.72E-33 |
| JUN | ENSG00000177606 | 2.58 | 0.20 | 6.83E-37 | 3.16E-33 |
| FOSB | ENSG00000125740 | 3.23 | 0.26 | 5.36E-35 | 1.49E-31 |
| RP11-94M14.2 | ENSG00000224750 | -1.60 | 0.13 | 5.38E-35 | 1.49E-31 |
| SGK1 | ENSG00000118515 | 3.27 | 0.28 | 3.35E-31 | 7.74E-28 |
| PPP1R15A | ENSG00000087074 | 1.55 | 0.14 | 1.12E-28 | 2.23E-25 |
| SAT1 | ENSG00000130066 | 1.69 | 0.16 | 1.43E-27 | 2.48E-24 |
| METTL12 | ENSG00000214756 | 1.88 | 0.18 | 7.00E-26 | 1.08E-22 |
| IER5 | ENSG00000162783 | 1.30 | 0.13 | 5.53E-25 | 7.68E-22 |
| HSPA1B | ENSG00000204388 | 1.38 | 0.14 | 2.49E-24 | 3.14E-21 |
| IFIT2 | ENSG00000119922 | 1.24 | 0.13 | 6.29E-23 | 7.28E-20 |
| RGS2 | ENSG00000116741 | 1.96 | 0.20 | 1.56E-22 | 1.66E-19 |
| TRIB3 | ENSG00000101255 | 1.29 | 0.14 | 1.13E-21 | 1.12E-18 |
| ASS1 | ENSG00000130707 | -0.71 | 0.08 | 2.70E-21 | 2.34E-18 |
| PTPRE | ENSG00000132334 | 0.71 | 0.08 | 2.53E-21 | 2.34E-18 |
| ID2 | ENSG00000115738 | 1.55 | 0.17 | 2.88E-20 | 2.35E-17 |
| ACTG1P22 | ENSG00000271615 | -1.10 | 0.12 | 1.74E-19 | 1.35E-16 |
| RHOB | ENSG00000143878 | 1.24 | 0.14 | 3.72E-19 | 2.72E-16 |
| E2F1 | ENSG00000101412 | -1.08 | 0.12 | 4.41E-19 | 3.06E-16 |
| FAM69C | ENSG00000187773 | -0.98 | 0.11 | 1.75E-18 | 1.16E-15 |
| FAM129A | ENSG00000135842 | 1.00 | 0.12 | 2.55E-18 | 1.61E-15 |
| SH3PXD2A | ENSG00000107957 | 0.82 | 0.09 | 3.31E-18 | 2.00E-15 |
| SOCS2 | ENSG00000120833 | -0.83 | 0.10 | 4.19E-18 | 2.42E-15 |
| ENC1 | ENSG00000171617 | 0.85 | 0.10 | 8.01E-18 | 4.45E-15 |
| POLR2L | ENSG00000177700 | -0.82 | 0.10 | 1.26E-17 | 6.75E-15 |
| ACTB | ENSG00000075624 | -0.70 | 0.08 | 5.58E-17 | 2.87E-14 |
| RRM2 | ENSG00000171848 | -0.72 | 0.09 | 9.71E-17 | 4.82E-14 |
| ATP5J2 | ENSG00000241468 | -0.87 | 0.11 | 2.06E-16 | 9.86E-14 |
| ASF1B | ENSG00000105011 | -0.86 | 0.11 | 2.70E-16 | 1.21E-13 |
| NUP62 | ENSG00000213024 | -0.86 | 0.11 | 2.65E-16 | 1.21E-13 |
| ARHGEF4 | ENSG00000136002 | 0.88 | 0.11 | 3.63E-16 | 1.57E-13 |
| PKMYT1 | ENSG00000127564 | -1.15 | 0.14 | 9.87E-16 | 4.15E-13 |
| MCM2 | ENSG00000073111 | -0.80 | 0.10 | 1.40E-15 | 5.72E-13 |
| HMX2 | ENSG00000188816 | -0.87 | 0.11 | 1.51E-15 | 5.99E-13 |
| PCNA | ENSG00000132646 | -0.88 | 0.11 | 1.59E-15 | 6.14E-13 |
| LINC01623 | ENSG00000225595 | 2.41 | 0.30 | 2.22E-15 | 8.33E-13 |
| ANKRD30BL | ENSG00000163046 | 1.01 | 0.13 | 3.46E-15 | 1.26E-12 |
| TK1 | ENSG00000167900 | -0.94 | 0.12 | 7.09E-15 | 2.52E-12 |
| GADD45B | ENSG00000099860 | 1.01 | 0.13 | 7.56E-15 | 2.63E-12 |
| TNFSF4 | ENSG00000117586 | 0.82 | 0.11 | 7.86E-15 | 2.66E-12 |
| HEXIM1 | ENSG00000186834 | 1.01 | 0.13 | 3.20E-14 | 1.06E-11 |
| MCM4 | ENSG00000104738 | -0.89 | 0.12 | 3.45E-14 | 1.11E-11 |
| CFL1 | ENSG00000172757 | -0.59 | 0.08 | 3.52E-14 | 1.11E-11 |
| UNG | ENSG00000076248 | -0.95 | 0.13 | 4.64E-14 | 1.43E-11 |
| GINS2 | ENSG00000131153 | -0.93 | 0.12 | 6.94E-14 | 2.09E-11 |
| TALDO1 | ENSG00000177156 | -0.71 | 0.10 | 8.67E-14 | 2.56E-11 |
| CDT1 | ENSG00000167513 | -1.00 | 0.14 | 9.50E-14 | 2.75E-11 |
| RSAD2 | ENSG00000134321 | 0.95 | 0.13 | 1.07E-13 | 3.02E-11 |
| ACTBP2 | ENSG00000213763 | -1.19 | 0.16 | 1.47E-13 | 4.09E-11 |
| MCM10 | ENSG00000065328 | -0.70 | 0.10 | 2.02E-13 | 5.51E-11 |
| CXCL8 | ENSG00000169429 | 1.97 | 0.27 | 2.47E-13 | 6.59E-11 |
| BIRC3 | ENSG00000023445 | 2.48 | 0.34 | 3.26E-13 | 8.54E-11 |
| ERH | ENSG00000100632 | -0.62 | 0.09 | 3.73E-13 | 9.59E-11 |
| RPS15 | ENSG00000115268 | -0.50 | 0.07 | 4.11E-13 | 1.04E-10 |
| PFN1 | ENSG00000108518 | -0.63 | 0.09 | 4.39E-13 | 1.09E-10 |
| TOB1 | ENSG00000141232 | 1.40 | 0.19 | 4.86E-13 | 1.18E-10 |
| DDIT4 | ENSG00000168209 | 0.81 | 0.11 | 5.56E-13 | 1.33E-10 |
| MDK | ENSG00000110492 | -0.56 | 0.08 | 1.11E-12 | 2.60E-10 |
| MCM6 | ENSG00000076003 | -0.73 | 0.10 | 1.18E-12 | 2.73E-10 |
| CHAC1 | ENSG00000128965 | -0.90 | 0.13 | 1.22E-12 | 2.79E-10 |
| DMGDH | ENSG00000132837 | 1.91 | 0.27 | 1.69E-12 | 3.79E-10 |
| DTL | ENSG00000143476 | -0.84 | 0.12 | 1.83E-12 | 4.04E-10 |
| DDIT4L | ENSG00000145358 | -0.66 | 0.09 | 1.88E-12 | 4.08E-10 |
| CD52 | ENSG00000169442 | -0.70 | 0.10 | 2.35E-12 | 5.02E-10 |
| MPO | ENSG00000005381 | -0.77 | 0.11 | 2.56E-12 | 5.37E-10 |
| CNN2 | ENSG00000064666 | -0.78 | 0.11 | 3.08E-12 | 6.39E-10 |
| FTH1 | ENSG00000167996 | 0.60 | 0.09 | 3.19E-12 | 6.51E-10 |
| CITED2 | ENSG00000164442 | 0.90 | 0.13 | 3.27E-12 | 6.58E-10 |
| PRKCSH | ENSG00000130175 | -0.57 | 0.08 | 3.57E-12 | 7.08E-10 |
| FMNL3 | ENSG00000161791 | 0.77 | 0.11 | 3.83E-12 | 7.49E-10 |
| ELFN1 | ENSG00000225968 | -0.77 | 0.11 | 3.90E-12 | 7.52E-10 |
| CDC25A | ENSG00000164045 | -0.88 | 0.13 | 5.33E-12 | 1.01E-09 |
| PALM | ENSG00000099864 | -0.78 | 0.11 | 6.23E-12 | 1.17E-09 |
| ND3 | ENSG00000198840 | -0.56 | 0.08 | 6.71E-12 | 1.24E-09 |
| TNFRSF10B | ENSG00000120889 | 0.59 | 0.09 | 1.19E-11 | 2.17E-09 |
| RGS16 | ENSG00000143333 | 2.07 | 0.31 | 1.44E-11 | 2.60E-09 |
| RANBP1 | ENSG00000099901 | -0.72 | 0.11 | 1.81E-11 | 3.22E-09 |
| MCM7 | ENSG00000166508 | -0.68 | 0.10 | 1.94E-11 | 3.41E-09 |
| BTG1 | ENSG00000133639 | 0.67 | 0.10 | 2.01E-11 | 3.49E-09 |
| RND1 | ENSG00000172602 | 2.36 | 0.35 | 2.27E-11 | 3.89E-09 |
| POLD1 | ENSG00000062822 | -0.67 | 0.10 | 2.36E-11 | 3.99E-09 |
| TUBAP2 | ENSG00000214391 | -1.20 | 0.18 | 2.58E-11 | 4.32E-09 |
| IER5L | ENSG00000188483 | 1.81 | 0.27 | 3.22E-11 | 5.32E-09 |
| ASS1P1 | ENSG00000220517 | -1.33 | 0.20 | 3.32E-11 | 5.42E-09 |
| CYBA | ENSG00000051523 | -0.80 | 0.12 | 3.49E-11 | 5.57E-09 |
| ZMYND19 | ENSG00000165724 | -0.59 | 0.09 | 3.48E-11 | 5.57E-09 |
| TYMS | ENSG00000176890 | -0.76 | 0.12 | 3.56E-11 | 5.62E-09 |
| SNAI1 | ENSG00000124216 | 2.23 | 0.34 | 3.87E-11 | 6.04E-09 |
| TUBB | ENSG00000196230 | -0.50 | 0.08 | 4.24E-11 | 6.54E-09 |
| CD82 | ENSG00000085117 | 1.25 | 0.19 | 5.05E-11 | 7.70E-09 |
| CDC45 | ENSG00000093009 | -0.74 | 0.11 | 5.45E-11 | 8.22E-09 |
| MYBL2 | ENSG00000101057 | -0.64 | 0.10 | 5.96E-11 | 8.90E-09 |
| TMSB10 | ENSG00000034510 | -0.55 | 0.08 | 7.73E-11 | 1.14E-08 |
| NOC4L | ENSG00000184967 | -0.75 | 0.12 | 7.97E-11 | 1.16E-08 |
| CHAF1A | ENSG00000167670 | -0.75 | 0.12 | 8.46E-11 | 1.22E-08 |
| MYC | ENSG00000136997 | -0.58 | 0.09 | 8.78E-11 | 1.24E-08 |
| MZT2B | ENSG00000152082 | -0.81 | 0.13 | 8.75E-11 | 1.24E-08 |
| UTRN | ENSG00000152818 | 0.54 | 0.08 | 9.86E-11 | 1.38E-08 |
| OASL | ENSG00000135114 | 4.05 | 0.63 | 1.09E-10 | 1.51E-08 |
|  | **…** |  |  |  |  |
| SERPINE1 | ENSG00000106366 | 1.39 | 0.74 | 5.89E-02 | NA |

**Table S3. Top-100 *RP11-137H2.4* silencing-specific deregulated genes upon 24h DMSO treatment.**

| **Gene symbol** | **Gene name** | **Log2 Fold-Change** | **Log2 Fold-change SE** | **P-value** | **Adjusted P-value** |
| --- | --- | --- | --- | --- | --- |
| EFEMP1 | ENSG00000115380 | -2.67 | 0.07 | 0.00E+00 | 0.00E+00 |
| NRAS | ENSG00000213281 | -1.91 | 0.05 | 1.69E-306 | 1.45E-302 |
| SOCS2 | ENSG00000120833 | 1.71 | 0.06 | 6.12E-185 | 3.51E-181 |
| ACTG1P22 | ENSG00000271615 | -1.87 | 0.07 | 2.89E-172 | 1.24E-168 |
| KCNJ2 | ENSG00000123700 | 2.01 | 0.08 | 4.52E-146 | 1.55E-142 |
| OSR2 | ENSG00000164920 | 2.25 | 0.09 | 1.09E-145 | 3.14E-142 |
| RERGL | ENSG00000111404 | -2.19 | 0.09 | 4.14E-125 | 1.02E-121 |
| EPHA7 | ENSG00000135333 | 1.45 | 0.07 | 4.66E-106 | 1.00E-102 |
| LINC00426 | ENSG00000238121 | -1.17 | 0.06 | 7.55E-95 | 1.44E-91 |
| NTRK1 | ENSG00000198400 | 2.10 | 0.10 | 4.66E-93 | 8.01E-90 |
| AGR2 | ENSG00000106541 | -3.34 | 0.17 | 1.77E-84 | 2.76E-81 |
| PTGDR | ENSG00000168229 | -2.60 | 0.14 | 3.30E-83 | 4.73E-80 |
| RP11-94M14.2 | ENSG00000224750 | -1.41 | 0.08 | 1.30E-71 | 1.72E-68 |
| HOMER1 | ENSG00000152413 | 1.14 | 0.06 | 9.92E-71 | 1.22E-67 |
| BMP2 | ENSG00000125845 | 1.77 | 0.10 | 1.20E-70 | 1.38E-67 |
| LST1 | ENSG00000204482 | -1.24 | 0.07 | 4.13E-70 | 4.44E-67 |
| NUP43 | ENSG00000120253 | -0.92 | 0.05 | 1.27E-69 | 1.29E-66 |
| JTB | ENSG00000143543 | -1.14 | 0.07 | 3.49E-68 | 3.33E-65 |
| HIST1H1T | ENSG00000187475 | 1.65 | 0.10 | 4.17E-67 | 3.77E-64 |
| HBEGF | ENSG00000113070 | 1.99 | 0.12 | 1.59E-66 | 1.37E-63 |
| KCNJ16 | ENSG00000153822 | 1.49 | 0.09 | 2.90E-65 | 2.38E-62 |
| FNDC5 | ENSG00000160097 | 1.69 | 0.10 | 2.84E-64 | 2.22E-61 |
| RASSF2 | ENSG00000101265 | -0.96 | 0.06 | 1.51E-62 | 1.13E-59 |
| ARHGEF4 | ENSG00000136002 | -1.45 | 0.09 | 4.22E-62 | 3.02E-59 |
| GPRIN3 | ENSG00000185477 | 1.66 | 0.10 | 1.35E-57 | 9.28E-55 |
| ABLIM1 | ENSG00000099204 | -1.18 | 0.07 | 2.48E-57 | 1.64E-54 |
| RPS6KA4 | ENSG00000162302 | -1.04 | 0.07 | 9.36E-56 | 5.96E-53 |
| H1F0 | ENSG00000189060 | 0.90 | 0.06 | 1.02E-54 | 6.25E-52 |
| AOX2P | ENSG00000243478 | -1.43 | 0.09 | 1.29E-54 | 7.64E-52 |
| SLC38A5 | ENSG00000017483 | -0.90 | 0.06 | 5.45E-54 | 3.12E-51 |
| CACUL1 | ENSG00000151893 | -0.91 | 0.06 | 7.01E-52 | 3.89E-49 |
| JUNB | ENSG00000171223 | 1.18 | 0.08 | 5.02E-50 | 2.70E-47 |
| MYC | ENSG00000136997 | 0.82 | 0.06 | 3.60E-49 | 1.87E-46 |
| BACH2 | ENSG00000112182 | -0.93 | 0.06 | 5.71E-49 | 2.89E-46 |
| MME | ENSG00000196549 | -0.73 | 0.05 | 1.12E-48 | 5.50E-46 |
| SPRY2 | ENSG00000136158 | 0.92 | 0.06 | 9.90E-47 | 4.73E-44 |
| CLIC4 | ENSG00000169504 | -0.92 | 0.06 | 3.54E-46 | 1.65E-43 |
| DDIT4L | ENSG00000145358 | 0.99 | 0.07 | 1.41E-45 | 6.39E-43 |
| CD19 | ENSG00000177455 | -1.26 | 0.09 | 1.62E-45 | 7.14E-43 |
| RMND5A | ENSG00000153561 | -0.70 | 0.05 | 6.06E-45 | 2.61E-42 |
| PSD4 | ENSG00000125637 | -1.09 | 0.08 | 3.11E-44 | 1.31E-41 |
| FAM101B | ENSG00000183688 | -1.07 | 0.08 | 1.31E-43 | 5.36E-41 |
| SP4 | ENSG00000105866 | -1.00 | 0.07 | 2.61E-43 | 1.05E-40 |
| OSTC | ENSG00000198856 | -0.78 | 0.06 | 3.27E-43 | 1.28E-40 |
| SH3RF3 | ENSG00000172985 | -1.18 | 0.09 | 3.57E-43 | 1.36E-40 |
| FMNL3 | ENSG00000161791 | -0.99 | 0.07 | 6.51E-43 | 2.43E-40 |
| RFTN1 | ENSG00000131378 | -0.86 | 0.06 | 8.16E-43 | 2.99E-40 |
| FAM107B | ENSG00000065809 | -0.69 | 0.05 | 1.31E-42 | 4.71E-40 |
| TAOK3 | ENSG00000135090 | -0.96 | 0.07 | 4.04E-42 | 1.42E-39 |
| ZFP36L1 | ENSG00000185650 | 0.93 | 0.07 | 7.52E-42 | 2.59E-39 |
| SET | ENSG00000119335 | -0.86 | 0.06 | 1.57E-41 | 5.31E-39 |
| NYNRIN | ENSG00000205978 | 1.09 | 0.08 | 2.13E-41 | 7.03E-39 |
| FRG2C | ENSG00000172969 | -1.13 | 0.08 | 3.84E-41 | 1.24E-38 |
| SYNGR1 | ENSG00000100321 | -1.08 | 0.08 | 7.91E-41 | 2.52E-38 |
| FAM129A | ENSG00000135842 | -0.83 | 0.06 | 1.14E-40 | 3.56E-38 |
| QRSL1 | ENSG00000130348 | -0.72 | 0.05 | 2.06E-40 | 6.34E-38 |
| FAM69C | ENSG00000187773 | 1.05 | 0.08 | 6.56E-40 | 1.98E-37 |
| MAP1A | ENSG00000166963 | 0.75 | 0.06 | 8.13E-40 | 2.41E-37 |
| CCDC90B | ENSG00000137500 | -0.87 | 0.07 | 1.22E-39 | 3.57E-37 |
| TSPAN13 | ENSG00000106537 | -1.01 | 0.08 | 1.88E-39 | 5.39E-37 |
| BCL6 | ENSG00000113916 | -0.81 | 0.06 | 2.22E-39 | 6.26E-37 |
| CYB5R3 | ENSG00000100243 | -1.29 | 0.10 | 2.39E-39 | 6.62E-37 |
| RRAGD | ENSG00000025039 | 0.76 | 0.06 | 2.55E-39 | 6.95E-37 |
| GCC1 | ENSG00000179562 | -0.92 | 0.07 | 1.15E-38 | 3.09E-36 |
| ZMAT3 | ENSG00000172667 | -0.87 | 0.07 | 5.32E-38 | 1.41E-35 |
| TUBA4A | ENSG00000127824 | -1.01 | 0.08 | 5.43E-38 | 1.41E-35 |
| KPNA6 | ENSG00000025800 | -0.65 | 0.05 | 1.93E-37 | 4.95E-35 |
| UNC13A | ENSG00000130477 | 1.17 | 0.09 | 2.40E-37 | 6.07E-35 |
| PRDX3 | ENSG00000165672 | 0.69 | 0.05 | 2.86E-37 | 7.14E-35 |
| DOCK8 | ENSG00000107099 | -0.72 | 0.06 | 5.63E-37 | 1.38E-34 |
| JAG1 | ENSG00000101384 | -1.32 | 0.10 | 5.85E-37 | 1.42E-34 |
| STT3B | ENSG00000163527 | -0.57 | 0.05 | 1.88E-36 | 4.49E-34 |
| SETP14 | ENSG00000240489 | -0.93 | 0.07 | 3.46E-36 | 8.15E-34 |
| PCDH10 | ENSG00000138650 | 0.97 | 0.08 | 4.20E-36 | 9.76E-34 |
| IKZF2 | ENSG00000030419 | 0.68 | 0.06 | 7.10E-36 | 1.63E-33 |
| NA | ENSG00000272016 | -1.49 | 0.12 | 7.40E-36 | 1.67E-33 |
| N4BP2 | ENSG00000078177 | -0.73 | 0.06 | 8.36E-36 | 1.87E-33 |
| ESCO2 | ENSG00000171320 | -0.75 | 0.06 | 1.15E-35 | 2.53E-33 |
| BEST3 | ENSG00000127325 | -1.06 | 0.09 | 1.57E-35 | 3.42E-33 |
| FAM19A1 | ENSG00000183662 | -2.27 | 0.18 | 1.72E-35 | 3.70E-33 |
| SPTBN2 | ENSG00000173898 | 0.75 | 0.06 | 2.48E-35 | 5.26E-33 |
| RP11-94M14.3 | ENSG00000238291 | -1.40 | 0.11 | 2.66E-35 | 5.57E-33 |
| KB-208E9.1 | ENSG00000272733 | -0.84 | 0.07 | 3.56E-35 | 7.38E-33 |
| LRIG1 | ENSG00000144749 | -0.86 | 0.07 | 5.02E-35 | 1.03E-32 |
| SOCS2-AS1 | ENSG00000246985 | 1.45 | 0.12 | 6.84E-35 | 1.38E-32 |
| LTB | ENSG00000227507 | -0.75 | 0.06 | 1.19E-34 | 2.38E-32 |
| DENND3 | ENSG00000105339 | -0.76 | 0.06 | 1.39E-34 | 2.75E-32 |
| AMMECR1 | ENSG00000101935 | -0.83 | 0.07 | 1.85E-34 | 3.62E-32 |
| RP5-912I13.1 | ENSG00000260426 | -0.98 | 0.08 | 2.36E-34 | 4.55E-32 |
| LDLRAD4 | ENSG00000168675 | -0.73 | 0.06 | 5.49E-34 | 1.05E-31 |
| UNC79 | ENSG00000133958 | -0.77 | 0.06 | 1.30E-33 | 2.46E-31 |
| SLC9A9 | ENSG00000181804 | -0.97 | 0.08 | 1.73E-33 | 3.23E-31 |
| MAPKAPK2 | ENSG00000162889 | -0.63 | 0.05 | 2.30E-33 | 4.24E-31 |
| SORL1 | ENSG00000137642 | -0.62 | 0.05 | 2.47E-33 | 4.52E-31 |
| ATP1A3 | ENSG00000105409 | 1.19 | 0.10 | 4.02E-33 | 7.27E-31 |
| ASS1 | ENSG00000130707 | 0.74 | 0.06 | 1.55E-32 | 2.77E-30 |
| MAP1S | ENSG00000130479 | 0.98 | 0.08 | 5.49E-32 | 9.74E-30 |
| PGRMC1 | ENSG00000101856 | 0.65 | 0.06 | 8.13E-32 | 1.43E-29 |
| PCDH18 | ENSG00000189184 | -0.82 | 0.07 | 8.92E-32 | 1.55E-29 |
| PRKAR2A | ENSG00000114302 | -0.73 | 0.06 | 2.57E-31 | 4.42E-29 |
|  | **…** |  |  |  |  |
| JUN | ENSG00000177606 | 0.52 | 0.14 | 1.97E-04 | 1.56E-03 |
| FOS | ENSG00000170345 | 0.48 | 0.16 | 3.40E-03 | 1.72E-02 |
| SGK1 | ENSG00000118515 | 0.02 | 0.12 | 8.69E-01 | 9.36E-01 |
| SERPINE1 | ENSG00000106366 | 0.25 | 0.15 | 9.74E-02 | 2.38E-01 |
